# Deep Learning-Based Non-Contact IPPG Signal Blood Pressure Measurement Research

**DOI:** 10.3390/s23125528

**Published:** 2023-06-13

**Authors:** Hanquan Cheng, Jiping Xiong, Zehui Chen, Jingwei Chen

**Affiliations:** College of Physics and Electronic Information Engineering, Zhejiang Normal University, Jinhua 321000, China

**Keywords:** deep learning, non-contact blood pressure, image photoplethysmography

## Abstract

In this paper, a multi-stage deep learning blood pressure prediction model based on imaging photoplethysmography (IPPG) signals is proposed to achieve accurate and convenient monitoring of human blood pressure. A camera-based non-contact human IPPG signal acquisition system is designed. The system can perform experimental acquisition under ambient light, effectively reducing the cost of non-contact pulse wave signal acquisition while simplifying the operation process. The first open-source dataset IPPG-BP for IPPG signal and blood pressure data is constructed by this system, and a multi-stage blood pressure estimation model combining a convolutional neural network and bidirectional gated recurrent neural network is designed. The results of the model conform to both BHS and AAMI international standards. Compared with other blood pressure estimation methods, the multi-stage model automatically extracts features through a deep learning network and combines different morphological features of diastolic and systolic waveforms, which reduces the workload while improving accuracy.

## 1. Introduction

With the progress of technology and social development, people’s living standards are gradually improving, while the morbidity and mortality of cardiovascular diseases are increasing year by year [1]. Cardiovascular disease (CVD) is the most prevalent disease in the world [2], of which hypertension is the most important causative factor in CVD, and the lack of obvious symptoms in the early stages of hypertension leads to a low diagnosis rate of 46%. In addition, less than 33% of patients are able to control their disease with effective treatment [1].

Blood pressure (BP) is the lateral pressure acting on the blood vessel wall per unit area during the circulation of blood through the blood vessels around the body and is essential for maintaining blood circulation. As one of the important parameters to measure the health of the human body, it is not only used to monitor the health condition of the human body but also can be used for disease diagnosis and treatment, which has important observation value. One of the most important physiological parameters in the cardiovascular response to the state of the human body is arterial blood pressure, and its accurate, long-term dynamic monitoring assumes an extremely important role in the modern medical system [3]. The commercially available “gold standard” method of blood pressure measurement is the invasive method of measurement. Invasive measurement requires the insertion of a catheter and a monitoring probe into the heart or blood vessel cavity through the human body surface and the use of sensors to obtain blood pressure values [4]. However, the arterial cannulation method technique must be operated by professionals, is expensive, and because it requires insertion into a blood vessel, it has the potential to cause multiple complications, such as bleeding and infection, and thus is usually used only in special situations such as surgery and intensive care units and cannot be implemented for everyday needs in the home [5].

A non-invasive optical detection technique for physiological signal detection, called photoplethysmography (PPG) [6], has emerged as a contact detection method that usually uses a photosensitive sensor to achieve physiological signal measurement. PPG technology has been widely used in the current medical detection systems. However, PPG technology has some shortcomings because it is still a contact detection method, for example, the subject may feel uncomfortable due to the continuous close contact with the instrument, and some subjects such as patients with large burns, patients with sensitive skin, and other special populations cannot pass the application of this technology. In addition, some equipment operation requirements are high, still need professional personnel to achieve the operation, and cannot meet the daily physiological monitoring needs. With the continuous improvement of people’s living standards, people hope to find a more convenient, convenient, and sustainable way to monitor blood pressure.

**Figure 1:**
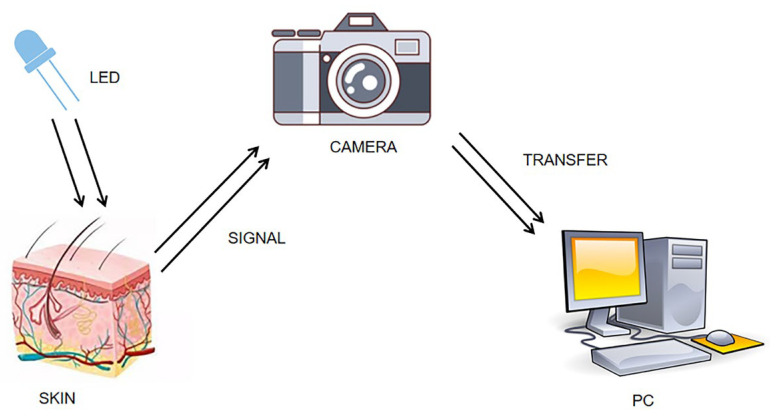
Diagram of IPPG signal acquisition experiment.

IPPG is a non-contact optical detection technique [7]. When a heartbeat causes blood to flow, it causes periodic changes in blood volume in the blood vessels. Ting et al. [8] proposed the concept of the IPPG technique in 2000. They performed near-infrared light irradiation skin experiments, and when the reflected light after the filter is imaged on a charge-coupled chip, each frame image is saved, followed by locating the region at the same location in the frame image as the ROI region, and by calculating the gray-scale mean value of each ROI region, and then depicting the obtained time series as the corresponding waveform curve, the IPPG signal reflecting the physiological condition of the pulse can finally be obtained; Figure 1 shows the schematic diagram of the experimental setup.

The periodic signal, like IPPG, cannot be recognized by the naked eye, but the face video can be captured by imaging devices such as industrial cameras, webcams, and cell phone lenses, from which the IPPG signal can be extracted. In addition, because different components within the blood have different light absorption properties, the selected specific wavelength of the applied light source can achieve the measurement of different physiological parameters, such as heart rate [9], blood oxygen saturation [10], blood pressure [11], and heart rate variability [12].

Currently, many scholars have conducted some preliminary exploratory experiments on non-contact blood pressure (NCBP) measurement [13,14,15]. Sugita et al. [16] proposed a non-contact blood pressure measurement method, which calculates two pulse wave signals in the same cycle of both signals by collecting them at different parts of the body and conduction times to estimate blood pressure. Their study showed that PTT had a higher correlation with systolic blood pressure. However, their experimental conditions could not be implemented in real-life scenarios. Jain et al. [17] proposed a method to estimate arterial blood pressure values using a regression model. This method was able to detect sudden changes in blood pressure due to physiological activity, which provides a basis for detecting sudden changes in blood pressure. Shao et al. [18] and Takahashi [19] achieved blood pressure prediction by pulse wave conduction times obtained from the face and palm regions. In their work, a tracking algorithm was introduced to handle the motion artifacts in the signal. However, they needed to photograph multiple parts of the human body simultaneously to obtain reliable PTT, and they used expensive camera equipment, which was not very practical. Hong et al. [20] used optical skin imaging to extract pulse wave features and achieved training and prediction by building an artificial neural network model. Additionally, many experiments were conducted in the study to verify the correlation between blood flow and blood pressure in the facial pulsatile system. However, the experimental dataset they used contained only normal blood pressure and only artificial neural network models were used for the experiments. In addition to this, the system requires an additional external light source for its operation.

Deep neural network-based blood pressure estimation algorithms, which are implemented by feeding features or waveforms to neural networks, are one of the main directions of current research on continuous noninvasive blood pressure monitoring. Compared with machine learning-based measurement methods, deep learning models are more capable of learning for high-dimensional features and have a better fitting ability for complex nonlinear relationships. Some studies have used deep neural networks (DNN) to achieve blood pressure measurement through features extracted manually from pulse waveforms [21]. However, the study still did not get rid of the cuff. There are also studies that acquire multiple human physiological signals simultaneously to achieve blood pressure estimation. However, acquiring multiple signals requires more acquisition equipment and greater effort [22]. Slapnivcar et al. [23] used the ResNet model to perform blood pressure prediction experiments on a large public dataset, and the final experiment achieved good results. Djamaleddine et al. [24] proposed a method for blood pressure measurement based on pulse wave feature parameters. They proposed several different feature extraction methods, but the method required a lot of human work in the preliminary preparation to extract the features. Wu et al. [25] implemented the fusion of pulse waveform features and personal information features and constructed a DNN model with multiple hidden layers to predict blood pressure by ECG signals and pulse wave signals, but the method required the simultaneous acquisition of ECG signal and pulse wave signal simultaneously, which is cumbersome to operate.

Current machine learning-based methods are highly accurate and fast, but require manual feature extraction, and inaccurate feature extraction can seriously affect the measurement results for noisy signals. Neural networks have been proven to perform well in many fields and have been applied to blood pressure estimation, however, due to the simplicity of the pulse waveform, current studies are still unable to accurately apply valid waveform features, and their results are less accurate and more laborious.

In order to overcome the above-mentioned problems, we investigated a deep learning-based algorithm for non-contact blood pressure estimation. A camera-based non-contact IPPG signal acquisition system is designed, and the first open-source IPPG signal and blood pressure dataset are constructed by capturing video of multiple volunteers’ faces as well as synchronized blood pressure data, and a multi-stage deep neural network is designed for blood pressure estimation to correlate the dynamic relationship between systolic and diastolic blood pressure to finally achieve z-accurate blood pressure measurement.

## 2. Materials and Methods

**Figure 2:**
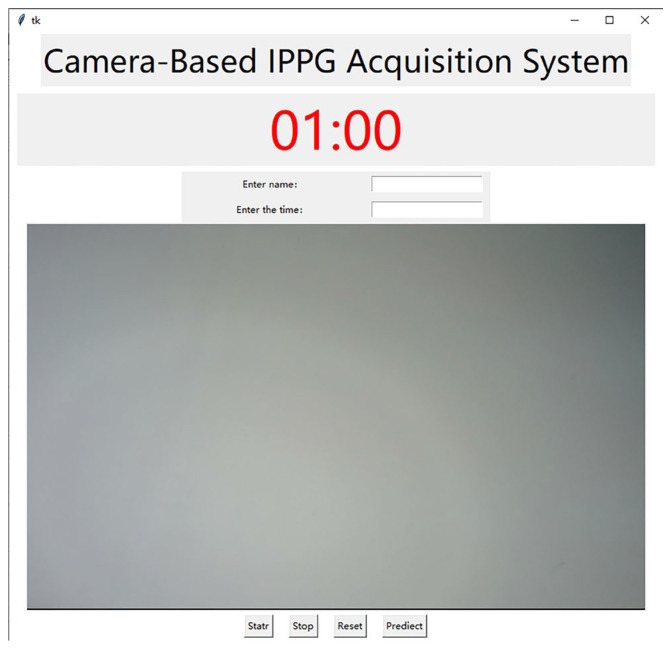
Interface of signal acquisition system.

In order to achieve better face video recording and signal acquisition, this project has developed a camera-based IPPG signal acquisition system, which is capable of capturing the images captured by the camera and displaying them to the system interface synchronously to facilitate the adjustment of the pose during recording and adding a timer recording function to meet the needs of different video recording durations with convenient operation. The interface of this acquisition system is shown in Figure 2. The specific process of face video capture includes the following steps:

(1) Use an external camera to capture the face video in a stationary state with stable ambient light and no other interference.
(2) Extract the IPPG signal from the region of interest of the face video. Face recognition and key point location are performed on the frame images, and the ROI region with the most suitable effect for extracting IPPG signal is selected through comparison experiments. The three channels of RGB are separated from the frame images in the ROI region, and then the mean value of the pixel intensity of the green channel in the ROI region is calculated frame by frame, and finally, a fixed-length pixel intensity change curve is obtained as the original pulse wave signal and saved.
(3) Signal pre-processing. The extracted pulse wave signal is wavelet transformed to remove the baseline drift, and then Butterworth bandpass filter is used to remove the high-frequency noise caused by the device itself to make the whole waveform smoother.

**Figure 3:**
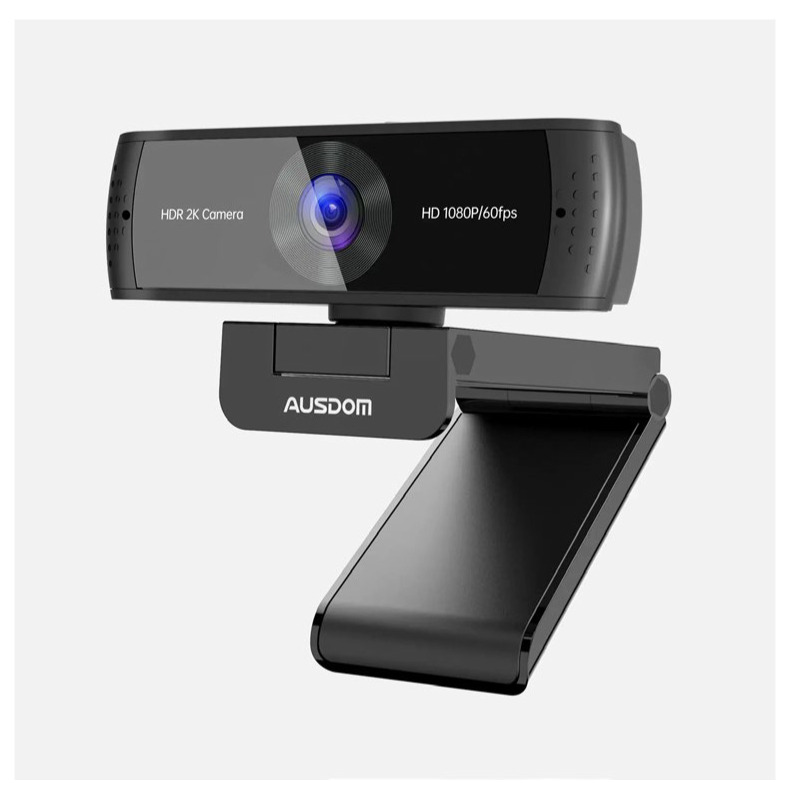
Aston camera.

Most of the current studies on non-contact blood pressure measurement use expensive industrial cameras or high-definition digital cameras, which leads to high experimental costs, is not conducive to equipment deployment and product promotion, and cannot meet the demand for non-contact blood pressure measurement in daily home life. The earliest common camera-based physiological parameter detection method was proposed by Poh et al., in 2010 [26], and although their experiments successfully measured heart rate, the camera used had a low frame rate and poor imaging quality and was less accurate in measuring other physiological parameters such as blood oxygen saturation and heart rate variability. Therefore, the Athlon AW651 computer camera (shown in Figure 3) produced by Shenzhen Asdun Cloud Technology Co., Ltd., Shenzhen, China was selected as the camera device for this experiment. The camera supports both 2K/30FPS and 1080P/60FPS recording modes, while it can restore HDR high dynamic images and present finer color differences, with a viewing angle of 75°, no distortion of faces and scenes during recording, and a sensitivity of −36 dB + 2 dB, which fully meets the requirements of the experimental signal acquisition system.

The laptop model used for the experiments is Legin R7000 2020.

(1) Processor: AMD Ryzen 5 4600H with Radeon Graphics (AMD, Santa Clara, CA, USA) 3.00 GHz.
(2) RAM: 16 GB.
(3) Operating system: Windows 10, 64 bit.

When the face video acquisition is completed using the camera, a series of operations such as ROI region selection, IPPG signal extraction, and processing need to be completed on the computer terminal.

The blood pressure collection device used for the experiment was the Yuyue YE670A. The sphygmomanometer uses an LCD LCD soft backlit screen to clearly identify blood pressure readings and uses a SOC chip and upgraded DFFA dual filtering algorithm for more accurate measurement results, in line with ESH European Society of Hypertension and AAMI American Association for the Advancement of Medical Devices standards.

When the video recording is completed, the system will first perform face recognition on the video, and if the face cannot be correctly recognized, the face video will be re-recorded; if it can be correctly recognized, the captured video will be saved, and used in the subsequent research. The algorithm used in this system is the Dlib face recognition algorithm, which has high accuracy and real-time performance and is a face recognition tool based on C++ and can be used in Python. The system uses a loaded Dlib face recognition model to locate face key points on separated frames. The model locates 68 key points, including organs such as mouth, nose, eyes, and the overall face contour. The face size can be calculated from the location information of the key points and the localization of the ROI region is completed.

The IPPG technology is developed based on the Lambert–Bier law and light scattering theory [27,28]. Lambert–Bier’s law is described as the relationship between the transmitted light intensity and the reflected light intensity when light of wavelength λ is irradiated to a certain solution as shown in Equation (1):

(1) $$I=I_{0}e^{-\varepsilon (\gamma )CL}$$

where ε(γ) is the absorption coefficient, C is the medium concentration, and L is the distance light travels through the substance. When light hits the skin tissue, the light intensity changes as the blood volume in the blood vessels changes periodically, and the reflected light intensity contains much of the physiological information of the body.

**Figure 4:**
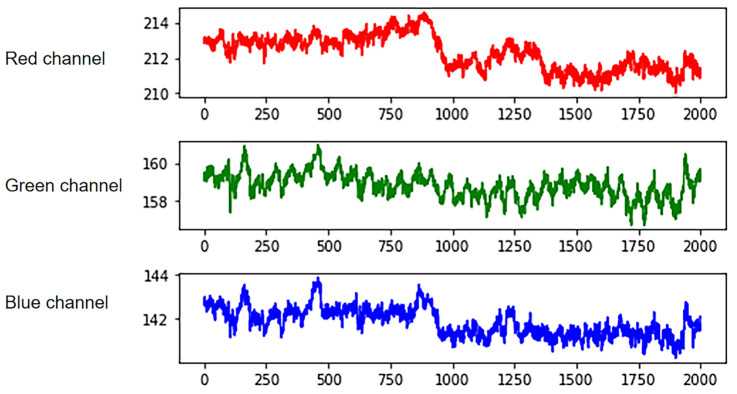
RGB three-channel separation.

The IPPG signal is obtained by a weighted average of the pixel values in the area. The calculation formula is shown below:

(2) $$S\left(t\right)=\frac{\sum_{i=1}^{W}\sum_{j=1}^{H}S\left(i,j\right)}{W\times H}$$

where t is the number of frame sequences, and W and H are the width and height of the region of interest. These videos are decomposed into frame pictures, and then the three components of RGB channels of each frame are extracted from the videos. Obtain the signal amplitude by calculating the pixel average. Finally, the three-channel IPPG signal is obtained by Equation (3), as shown in Figure 4. Curves of different colors represent signals extracted from different channels. The optical absorption property of hemoglobin reaches its maximum when the wavelength of light is in the range of 500 nm–600 nm, which corresponds to the green channel signal [29]. Meanwhile, the green channel signal has the least noise interference, so all subsequent studies extracted the IPPG signal by the green channel signal.

There is a lot of noise in the original IPPG signal, mainly including the power frequency noise of the equipment and the baseline drift caused by the movement of the face. In this experiment, the denoising operation is realized by wavelet transform and Butterworth bandpass filter, and the noise in the signal is removed.

The wavelet threshold denoising method mainly analyzes and utilizes the information of the respective signal characteristics of the actual signal and the interference noise and performs the denoising process based on the different characteristics of the two when wavelet decomposition is performed. The noise reduction can be achieved by separating the effective signal from the noisy signal while maintaining the original characteristics of the actual signal to the maximum extent possible, and without destroying the signal needed for the experiment. The discrete wavelet transform of a one-dimensional signal is shown in Equation (3):

(3) $$F_{\psi }\left(x,y\right)=x_{0}^{-\frac{j}{2}}\int_{-\infty }^{+\infty }S_{iPPG}\left(t\right)\psi \left(x_{0}^{-j}t-y_{0}k\right)dt$$

where x denotes the scale factor, y denotes the translation factor, j and k are the parameters of the discrete x and y, respectively. In practical applications, generally set $x_{0}=2$, $y_{0}=1$, the above formula is transformed into a binary wavelet.

(4) $$\psi_{j,k}(t)=2^{-\frac{j}{2}}\psi (2^{-j}t-k)$$

The wavelet transform can be decomposed to different degrees according to the different characteristics of the signal to be denoised, in order to achieve the best signal denoising effect. In the practical application of signal denoising, the selection of wavelet transform scale is related to the sampling frequency.

**Figure 5:**
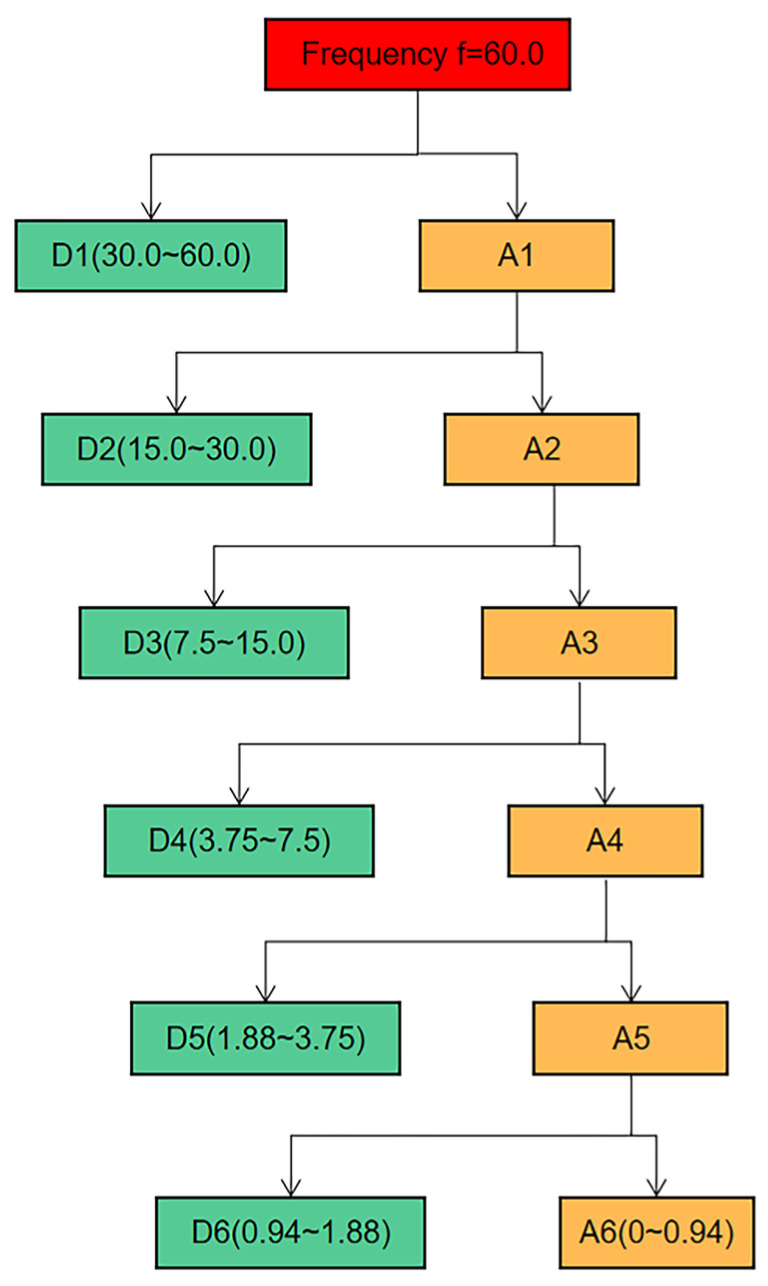
Signal six-scale decomposition schematic.

In this paper, the IPPG signal is taken as an example to perform six-scale wavelet decomposition, and the frequency ranges of each scale decomposition are shown in Figure 5. The approximation coefficients of each layer in the figure are used to represent, and the detail coefficients are used to represent, and the study shows that the baseline drift phenomenon occurs in the low-frequency region, concentrated in, and on. When recording the video, the frame rate of the camera is set to 60 FPS, while the breathing frequency is between 0.2 and 0.8 Hz, which corresponds to the sixth layer of wavelet decomposition. Therefore, Sym6 was selected as the wavelet basis function for the experiment, and the signal was decomposed into six layers, where the low-frequency component in the sixth layer was considered as the baseline drift signal. The sixth layer low-frequency component of the wavelet signal is subtracted from the original signal to achieve the removal of the baseline drift.

**Figure 6:**
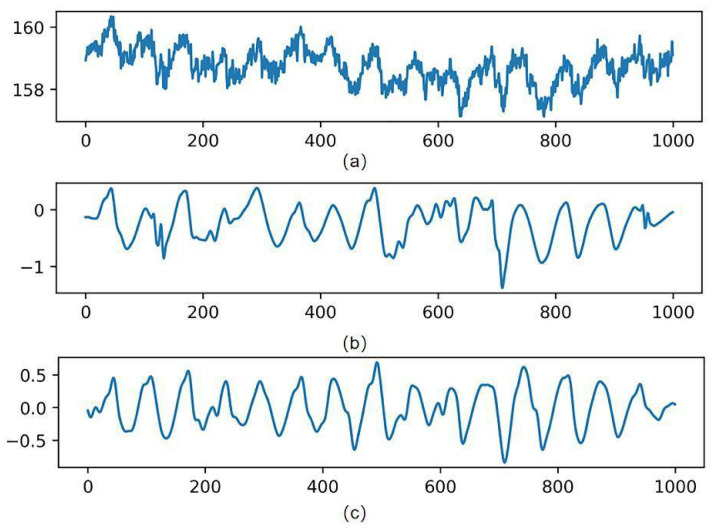
Signal processing results. (**a**) Is the original signal, (**b**) is the wavelet-transformed signal, and (**c**) is the band-pass filtered signal.

Studies have shown that the frequency range of the pulse wave is 0.7 Hz–6 Hz [30]. For the high-frequency noise caused by the circuit devices, the experiments were filtered using a fourth-order bidirectional Butterworth (Butterworth) low-pass filter with a cutoff frequency of 8 Hz and then a fourth-order bidirectional Butterworth high-pass filter to remove the low-frequency noise with a cutoff frequency of 0.6 Hz. The most significant specific of the Butterworth filter is the frequency response curve in the passband flat, which can make the filtered waveform smoother and retain the useful information in the IPPG signal to the maximum extent. By the above operation, the denoising of the IPPG signal is experimentally achieved. Figure 6 shows the process of the original IPPG signal processing.

For the selection of ROI regions, some studies choose the overall face region for the experiment. However, parts including the eyes and corners of the mouth can produce small motion artifacts due to breathing or subconscious movements. Such motion artifacts can affect the experimental results to a certain extent. Therefore, it is not advisable to select all the face areas. It is also considered that when conducting experiments on female subjects, many people’s hair will cover the forehead area, which will also have an impact on the photography. A large number of studies have shown that only some facial regions contain rich vascular information. Since the selection of ROI is directly related to the accuracy of subsequent signal extraction, it is extremely important to select the correct ROI containing rich vascular information for the study. Therefore, this section selects the ROI region with the best signal extraction effect by conducting comparison experiments with IPPG signals extracted from three regions: forehead, nose and cheeks, and mouth, respectively.

**Figure 7:**
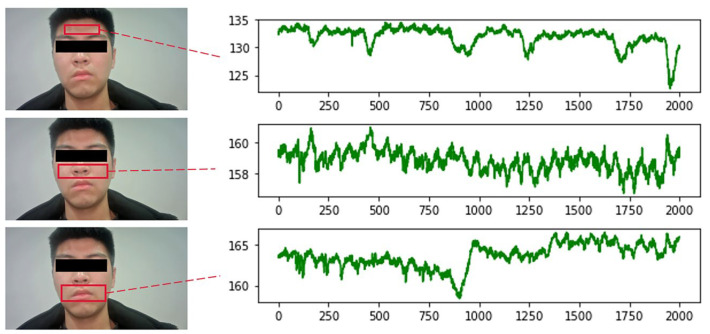
Comparison of different ROI signals.

The experiments were conducted by selecting 8 face videos of the subjects, extracting IPPG signals from three different ROI regions, drawing the mean green channel grayscale curves of different regions, and finding the most suitable ROI region through comparison experiments. The video recording duration is 60 s, the resolution is 1920 × 1080, the frame rate is 60 FPS, the natural light irradiation conditions are carried out, and the experimental subject is required to be in a calm state when recording. The pulse wave signals were extracted from three parts of the forehead, nose and cheeks, and mouth, respectively, for comparison experiments. Figure 7 shows the comparative experimental results of one of the experimenters.

From the above figure, it can be seen that the signal waveforms extracted from the cheek and nose regions are smoother and have less noise interference compared with the other two regions. There are two reasons for this result: firstly, this region is more stable and does not produce larger displacement due to human respiration, so there is less interference from motion artifacts; secondly, this region is rich in capillary distribution, which can better reflect physiological information. Therefore, this region was selected as the ROI region for extracting IPPG signals in all subsequent studies.

To ensure the accuracy of the experiment, a total of 115 volunteers aged 18 to 40 years old (including 74 males and 41 females) from the School of Physics and Electronic Information of Zhejiang Normal University and Zhejiang Yunpeng Technology Co. were recruited to participate in this study. All volunteers understood the experimental task description in advance and signed the informed consent form before the experiment, and none of them took cardiovascular disease-related drugs or had any known cardiovascular disease.

**Figure 8:**
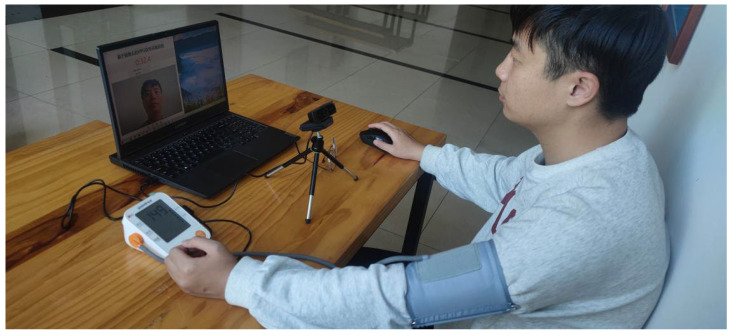
Data acquisition schematic.

The frame rate of the face video recorded in this experiment was 60 FPS, and the image resolution was 1920 × 1080. Usually, the human heart beats at 0.5–4 Hz per minute, so the frame rate of the camera used in the experiment fully met the demand. Before recording the video, the volunteers were asked to sit quietly for five minutes to maintain a stable physiological state, and then sit on a chair about 0.5 m in front of the camera in an environment with a stable light source to record the video. In order to ensure the reliability of the experiment, two time periods were chosen: morning and afternoon. A schematic diagram of the data acquisition process is shown in Figure 8.

**Figure 9:**
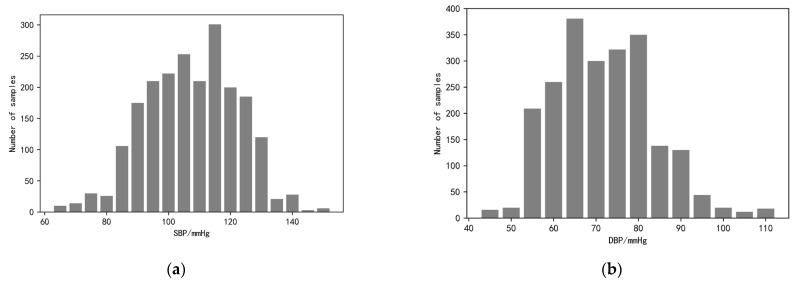
Histogram of blood pressure distribution. (**a**) The graph shows the distribution of systolic blood pressure data ranging from <70, 70 to 150, and >150; (**b**) the graph shows the distribution of diastolic blood pressure data ranging from <50, 50 to 110, and >110.

During the face video recording, the volunteers were asked to keep their heads as stable as possible and not to wear accessories that would obscure the facial region of interest to ensure that the pulse wave signals could be extracted correctly. The study also used a commercial sphygmomanometer, the Yuyue YE670A (which meets ESH as well as AAMI standards) to measure blood pressure and heart rate simultaneously and record them, with the signal pre-processing process as described previously. Finally, by collecting data from 115 volunteers, we constructed and open-sourced the first dataset for imaging-based photovolumetric pulse wave signals and simultaneous blood pressure—IPPG-BP (https://github.com/czh846567921/IPPG-BP/tree/main, accessed on 28 February 2023). The dataset contains 2120 pulse wave segments and their corresponding blood pressure data. To facilitate model training, the study classifies the blood pressure data into one category for every 5 mmHg, and the histogram of the blood pressure data distribution is shown in Figure 9. The study randomly selected data from 81 of 115 volunteers as the training set, while the data from the remaining 34 volunteers, which is now open, was used as the test set. The overall distribution of the data is shown in Table 1.

## 3. Methods

Compared with traditional blood pressure measurement methods based on machine learning, deep learning models can learn high-dimensional features and have stronger modeling capabilities for complex nonlinear systems. Currently, there are also many scholars studying the application of deep learning in blood pressure prediction work, but their studies then ignore the dynamic relationship that exists between systolic and diastolic blood pressure in each individual [29], and most of the studies estimating blood pressure by pulse wave signals show that diastolic blood pressure is more accurate than systolic blood pressure, which is due to the large fluctuations of systolic blood pressure, which leads to the complexity of the prediction process.

Based on the above, this study proposes a CNN + BiGRU multi-stage deep learning blood pressure estimation model that combines the dynamic relationship between diastolic and systolic blood pressure. The model consists of a two-stage network: the first stage is a convolutional neural network for extracting the morphological features of IPPG signals and performing preliminary blood pressure estimation; the second stage is a BiGRU network for training the time domain features of pulse waves, and finally building a multi-stage blood pressure estimation model. The method automatically extracts pulse wave features by deep learning model, fuses the dynamic relationship between diastolic and systolic blood pressure, and automatically processes complex and tedious calculations using a computer, reducing workload, and improving the intelligence and accuracy of the system.

**Figure 10:**
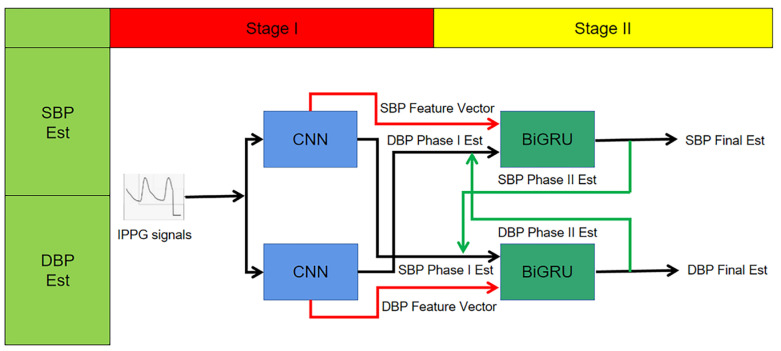
Multi-stage network structure schematic.

The block diagram of the multi-stage model is shown in Figure 10. The upper branch is used to estimate the systolic blood pressure, while the lower branch with used to estimate the diastolic blood pressure. Each trajectory consists of a two-stage deep neural network. Assigning two independent trajectories allows the network structure to extract features and cross-apply them to finally achieve blood pressure measurement.

In this study, the first neural network in each branch is a CNN. Two CNN models in one stage each contain four convolutional layers, and the output of the last convolutional layer is a signal feature vector, and the model output is a preliminary blood pressure estimate.

To improve the performance of this blood pressure estimation model, especially for estimating the SBP, the paper considers the correlation between the SBP and DBP, so that the extracted SBP features are extended by preliminary estimation of the DBP and vice versa; the extracted DBP feature vectors are extended by SBP estimation before being applied as input vectors to the next stage. This process is shown graphically in Figure 10 for the input of the second stage network. In other words:

Second stage SBP input vector = {SBP feature vector, DBP preliminary estimate}.
Second stage DBP input vector = {DBP feature vector, SBP preliminary estimate}.

The second neural network in each branch is a two-layer stacked BiGRU layer. The BiGRU network is able to track the long-term dependencies in its input sequence. Thus, the second stage estimates blood pressure based on current features and previous features. This capability improves the performance of the method by learning the temporal variation of the IPPG signal. In other words, since this stage uses all previous IPPG segments to estimate the current blood pressure, the estimated amount is a function of all the information previously provided to the network.

The final step to improve the prediction is to construct a feedback trajectory from the BiGRU output to the input, replacing the BP estimate from the first stage with a new BP estimate. Since the second stage is more accurate in estimating blood pressure, the experiment replaces the first-stage BP estimate with the BP estimate obtained from the second-stage calculation in the input of the BiGRU network to further improve the accuracy. This process will be repeated several times until the results stabilize.

**Figure 11:**
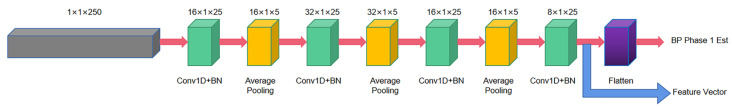
One-stage CNN structure diagram.

The first stage consists of two deep convolutional neural networks for performing the initial estimation of BP and extracting the feature vectors of systolic and diastolic BP. Each CNN contains four hidden convolutional layers and one Flatten layer (cf. Figure 11). The role of the Flatten layer is to transform the two-dimensional features into a one-dimensional sequence. The ReLU is used as the activation function. The output vector of the fourth convolutional layer containing 60 elements is used as is the extracted feature vector.

Considering the strong correlation between systolic and diastolic pressure, the experiments extend the systolic pressure feature vector by adding the one-stage estimate of diastolic pressure as an additional element, and vice versa. This output information containing 61 vectors was used as the extended vector input for the two-stage BiGRU model.

**Figure 12:**
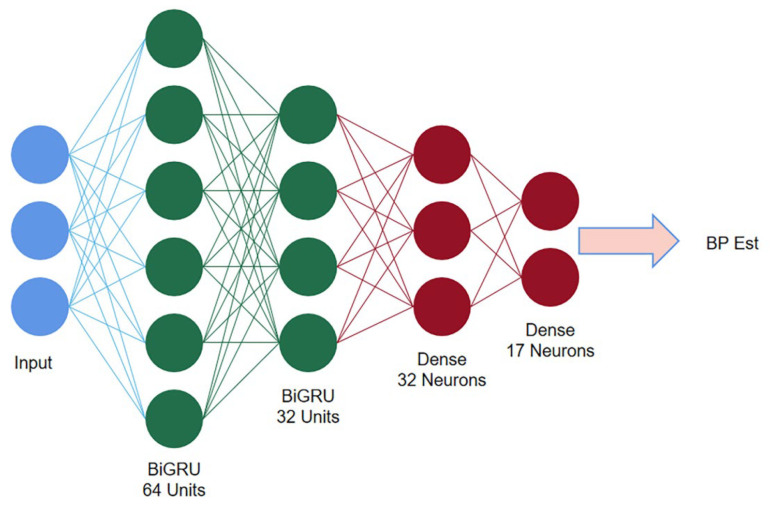
Second stage BiGRU structure diagram.

The second stage consists of two stacked BiGRU recurrent networks. The first BiGRU layer is set with 64 hidden neuron units, while the second layer is set with 32 (determined by experiment). The Softmax function is used as the activation function, the multivariate cross entropy (categorical crossentropy) is used as the loss function for training, and the learning rate is set to a fixed value of 0.001. The structure of the BiGRU network is shown in Figure 12.

## 4. Results

In this project, we used Anaconda for experimental simulation, 70% of the data were used for training and 30% of the data were used for testing, we used TensorFlow deep learning framework to build the network model, we used the default Adam as the optimizer, the data padding method was Padding = same, we also added a Dropout layer to prevent model overfitting, the parameter setting was 0.3. The first-order CNN model is trained with 500 iterations and the batch size is set to 32. The second-order BiGRU model is trained with 500 iterations and the batch size is set to 32, and the experiments are repeated several times for each model, using different random seeds each time to ensure the randomness of the data. The random seeds ensure that the ratio of the training set to the test set is 7:3 for each training, the experimental subjects in the training and test sets are independent of each other, and finally, the average of all predictions is taken as the final result. Finally, the performance is evaluated on the test set.

To ensure the accuracy of the experiments, a typical CNN model and a BiGRU model are also developed as comparison experiments on this topic. All these models have similar settings to the multi-stage model. To verify the accuracy of the models, three evaluation metrics, mean absolute error (MAE), standard deviation (STD), and mean deviation (ME), are used in this paper. In addition, the experiments also use regression curves and Bland–Altman plots for consistency analysis and regressivity analysis. Finally, the experimental results of this study were also compared with other related works. All analytical results below are based on data from the 30% validation set.

In this experiment, the experimental results of the two-stage model were compared with the multi-stage model. From Table 2, it can be seen that the MAE of SBP and DBP for the model of one-stage CNN are 8.24 mmHg and 6.42 mmHg, respectively, and the MAE of SBP and DBP for the two-stage BiGRU model are 7.57 mmHg and 5.76 mmHg, respectively. Finally, the MAE of SBP and DBP for the multi-stage model are 5.33 mmHg and 3.92 mmHg.

As can be seen from the table, the accuracy of the multi-stage model was higher than that of the separate-stage model. This indicates that fusing the dynamic relationship between systolic and diastolic blood pressure can effectively improve the accuracy of the blood pressure estimation model. The experimental results using the multi-stage model were analyzed subsequently.

## 5. Discussion

In this section, the experiments compare the prediction results of the multi-stage blood pressure model with two international metrics, the British Hypertension Society (BHS) and the American Association for the Advancement of Medical Devices (AAMI). The BHS classification criteria are based on the proportion of different mean absolute error ranges, which classify blood pressure measurements into three classes, A, B, and C, with error ranges of <5 mmHg, <10 mmHg, and <15 mmHg. Table 3 summarizes the performance of the multi-stage model based on the BHS criteria. As can be seen from the table, for SBP, MAE achieves grade B, grade A, and grade B on the three criteria of <5 mmHg, <10 mmHg, and <15 mmHg, respectively. For DBP, MAE obtained a grade of A on all three criteria, respectively. With the above results, it can be concluded that the experimentally proposed multi-stage model is in accordance with the BHS international standard.

The AAMI international standard differs from the BHS based on the mean deviation ME and standard deviation STD. AAMI requires that the ME and STD of DBP and SBP are less than 5 mmHg and 8 mmHg, respectively. From Table 4, it can be concluded that the ME of SBP and DBP are 2.02 mmHg and −1.38 mmHg, respectively, and the results are less than 5 mmHg, which is in accordance with the standard. The ME of SBP and the STD of DBP were 7.42 mmHg and 4.65 mmHg, respectively, both of which were also less than 8 mmHg. Therefore, it can be concluded that the experimentally proposed multi-stage model complies with the AAMI international standard.

**Figure 13:**
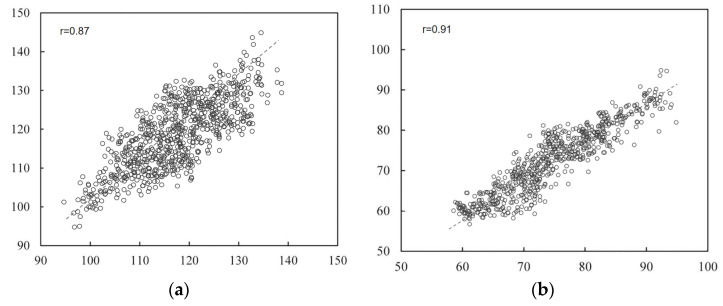
Regression analysis results. (**a**) Results of systolic blood pressure analysis (**b**) results of diastolic blood pressure analysis.

This section analyzes the prediction results of the multi-stage model through regression curves and Bland–Altman analysis plots. A scatter plot of the estimated blood pressure and the reference blood pressure is shown in Figure 13. From the figure, it can be concluded that for SBP, the correlation coefficient between the estimated blood pressure and the reference value is 0.87, and the data are evenly distributed. For DBP, the correlation coefficient between the estimated blood pressure and the reference value was 0.91, with concentrated data distribution. The scatter plot results indicate that the multi-stage model can achieve accurate blood pressure prediction.

**Figure 14:**
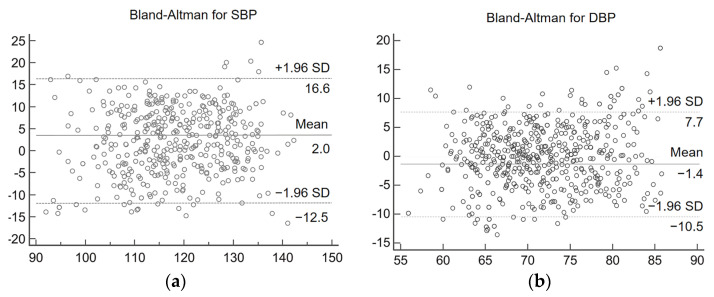
Consistency analysis graph. (**a**) Systolic blood pressure analysis results (**b**) diastolic blood pressure analysis results.

Bland–Altman analysis plots can be used to detect the consistency of the data. The Bland–Altman analysis plots of estimated blood values and reference blood pressure are shown in Figure 14. From Figure 14, it can be concluded that for the experimental results of SBP, 95.2% of the data were distributed within the consistency boundary. For the experimental results of DBP, 97.1% of the data were distributed within the consistency boundary. The results indicate that there is a high agreement between the estimated blood pressure and the reference blood pressure. In both analysis plots, the vast majority of the blood pressure estimates were accurate, but there were still a few estimates with large errors. This is because most of the volunteers selected for the experiment were relatively young and did not have cardiovascular-related diseases themselves, a condition that can lead to large errors in some abnormal blood pressure estimates. In addition, the multi-stage model proposed in this study is a general model, if personalized calibration for individuals is needed, more individual IPPG signal and blood pressure data need to be collected for model fine-tuning.

A large number of scholars have conducted research on blood pressure estimation based on pulse wave signals. Li et al. [31] trained time domain features by the SVM model to achieve blood pressure estimation. Meng and Kaiyang [32] extracted twenty-six features from IPPG signals for estimating blood pressure and trained them with four machine learning algorithms, and after comparing the experimental results, found that support vector regression was the best model for predicting blood pressure. Faust et al. [33] introduced features based on PPG amplitude to improve the long-term accuracy of blood pressure estimation. Fan et al. [34] proposed a non-contact BP estimation framework based on PTT. An adaptive Gaussian model for quadratic peak detection was proposed. By analyzing the basic features of IPPG, an adaptive Gaussian model was built to refine the shape of IPPG to obtain the adjusted PTT for deceitful BP. All the above studies require manual feature extraction, which can consume a lot of energy, while in the work of this paper, the BP estimation has high accuracy, while the pulse wave signal features are automatically extracted by the CNN model, which has better stability for practical application scenarios. Table 5 lists the experimental results of the studies related to blood pressure estimation, which are compared with the experimental results of this paper. The comparison results show that the multi-stage model designed in this experiment can achieve accurate blood pressure estimation by IPPG signal, the MAE of estimated SBP is the smallest, and the MAE and the smallest result of estimated DBP are extremely close on the dataset constructed by 115 volunteers, while the experimental subjects in this experiment are more numerous and the results are more generalizable.

Blood pressure estimation based on pulse wave features requires manual definition and calculation of feature values, and the dramatic changes in IPPG signals caused by vascular diseases, aging and physiological activities can affect the accuracy of manual feature extraction, or even prevent the features from being extracted correctly. The multi-stage blood pressure estimation model based on deep neural networks proposed in this study uses two convolutional neural networks in the first stage to extract the local morphological features of each IPPG segment and provide a preliminary estimate of blood pressure. Convolutional neural networks (CNNs) originate from the connectivity pattern between neurons in the visual cortex and use convolutional operations instead of the usual matrix multiplication. CNNs have fewer parameter connections to facilitate training than standard fully connected neural networks with the same number of layers [30] and are more accurate.

The second stage is two BiGRU networks, which are used to train the temporal features of the pulse wave signal. GRU can be considered a variant network of LSTM. The main improvement is the use of a new structure called “update gates” to replace the input and forgetting gates of the original LSTM. By optimizing the network structure, the training time is reduced without changing the memory function of neurons. The role of the update gate is twofold: first, to determine how much of the previous information is passed to the later neurons; second, to determine how much of the previous and current information is passed on. The reset gate, on the other hand, is responsible for determining how much of the past information is forgotten. The update gate also determines how much of the new information and how much of the old information is retained. The old and new information are in a complementary relationship; the more new information is retained, the less old information is retained, and vice versa. Additionally, BiGRU can establish the relationship between the information of the current moment and the features of the previous moment as well as learn the association between the feature information of the future moment and the information of the current moment. The BiGRU network consists of two GRUs with opposite directions, so BiGRU can transmit information both forward and backward so that the feature association between the future information and the past information is established, which solves the time series data features that are forgotten, thus improving the accuracy of blood pressure measurement. Notably, the study fully considers the dynamic relationship between systolic and diastolic blood pressure and establishes the hidden relationship between different features and blood pressure.

## 6. Conclusions

In this study, a camera-based non-contact facial signal acquisition system is designed. The system uses an external camera to record facial video, uses the Dlib algorithm to dynamically locate key points of the face, and selects regions of interest that contain rich vascular information so as to extract IPPG signals in ROI and preprocess them by wavelet transform and band-pass filtering. The first open-source dataset for IPPG and blood pressure signals, IPPG-BP, containing 115 volunteers with 2120 signal segments and corresponding systolic and diastolic blood pressure records, was constructed by a self-designed non-contact facial signal acquisition system. A multi-stage deep learning blood pressure estimation model based on CNN and BiGRU was designed. The multi-stage model uses a deep learning network to directly extract features from the pulse wave signal, solving the problem that traditional manual extraction of features is susceptible to interference from vascular diseases, aging, and physiological activities. The physiological relationship between diastole and systole is also considered, and the initial blood pressure estimates obtained using the diastolic and systolic feature vectors are then cross-fed into the BiGRU network in the second stage for data expansion, respectively. Experimental results show that the model can accurately achieve blood pressure estimation. The model also requires only a single IPPG signal to be acquired for blood pressure estimation, which is easily accessible and reduces workload while improving accuracy.

## Figures and Tables

**Table 1:** Blood pressure data distribution table.

	Individuals	Samples	SBP ME ± SD	DBP ME ± SD
Total sample	115	2120	116.4 ± 12.5	74.8 ± 9.7
Training set	81	1484	115.9 ± 12.6	74.3 ± 9.9
Test set	34	636	116.1 ± 12.3	74.2 ± 9.5

**Table 2:** Comparison of single model and multi-model results.

	SBP (mmHg)			DBP (mmHg)		
Models	MAE	STD	ME	MAE	STD	ME
CNN	8.24	9.31	3.98	6.42	6.83	2.11
BiGRU	7.57	8.83	−2.21	5.76	5.02	−1.89
Multi-stage model	5.33	7.42	−1.67	3.92	4.65	−1.08

**Table 3:** BHS standard comparison.

Error Percentage	<5 mmHg	<10 mmHg	<15 mmHg
Grade A	60%	85%	95%
Grade B	50%	75%	90%
Grade C	40%	65%	85%
SBP	53.2%	87.3%	94.6%
DBP	81.9%	93.1%	96.9%

**Table 4:** AAMI standards comparison.

	ME (mmHg)	STD (mmHg)
Estimation error	<5	<8
SBP	2.02	7.42
DBP	−1.38	4.65

**Table 5:** Comparison with the results of other research work.

Method	Data Type	Size	SBP MAE (mmHg)	DBP MAE (mmHg)
SVM [31]	PPG	7000 PPG samples	11.64	7.62
SVR [32]	IPPG	191 subjects	9.97	7.59
Regression [33]	PPG	27 subjects	5.49	4.01
Gaussian model [34]	IPPG	6 subjects	8.42	12.34
Ours	IPPG	115 subjects	5.33	4.02

## Data Availability

The data presented in https://github.com/czh846567921/IPPG-BP/tree/main, accessed on 28 February 2023.
